# P-838. The effect of transesophageal echocardiogram on clinical decision making for patients with Staphylococcus aureus blood stream infection

**DOI:** 10.1093/ofid/ofae631.1030

**Published:** 2025-01-29

**Authors:** Hiba Al Shaikhli, Mary Joyce B Wingler, Kayla R Stover, David A Cretella, Katie Barber, Jamie Wagner

**Affiliations:** University of Mississippi Medical Center, Houston, Texas; University of Mississippi Medical Center, Houston, Texas; University of Mississippi School of Pharmacy, Jackson, MS; University of Mississippi Medical Center, Houston, Texas; University of Mississippi, Jackson, MS; University of Mississippi School of Pharmacy, Jackson, MS

## Abstract

**Background:**

*Staphylococcus aureus* can lead to severe infections, including bloodstream infections, which are associated with high mortality. Infective endocarditis (IE) is a concerning complication of S. aureus bloodstream infections (SABSIs), which is ruled out by transesophageal echocardiogram (TEE). The COVID-19 pandemic led to a decrease in TEEs at the University of Mississippi Medical Center (UMMC) for patients with SABSIs. This study explores the impact of reduced TEE rates on clinical decisions for SABSI patients.

**Methods:**

This retrospective observational study assessed adult SABSI patients at UMMC, categorizing them into pre-COVID (admitted between 3/1/2018-3/11/2020) and COVID (admitted between 3/12/2020-3/1/2022) groups. A VIRSTA score, indicating IE risk and advocating TEE for high-risk patients (score ≥3), was calculated for patients. Primary outcomes included the rate of confirmed IE and antibiotic duration in pre-COVID and COVID groups. Secondary outcomes included hospital stay, correlation between VIRSTA score and TEE, 90-day mortality, and 60-day re-admission.

**Results:**

Among 214 patients, baseline characteristics were comparable between groups. The rate of confirmed IE (9.4% pre-COVID vs. 12.1% COVID; p = 0.66) and median antibiotic duration (28 days in both groups) showed no significant differences. However, mortality was more likely in the COVID group (22.2% vs 10.4%; p=0.019). Notably, high VIRSTA score patients were less likely to undergo TEE COVID (3.8% vs 18.5%; p = 0.008). There were no differences in other secondary outcomes.

**Conclusion:**

This retrospective study highlights the impact of reduced transesophageal TEE utilization during the COVID-19 pandemic on clinical decision-making for patients with SABSIs, including challenges in risk stratification based on VIRSTA score. Despite a decrease in TEE, there were no differences in confirmed IE diagnosis or duration of antibiotics, but mortality was higher in the COVID-19 group. Based on this study there is an opportunity to provide education related to VIRSTA score utility for risk stratification, which may preserve healthcare resources in select patients.

**Disclosures:**

**All Authors**: No reported disclosures

